# Quantification of minimal residual disease (MRD) in acute lymphoblastic leukemia (ALL) using amplicon-fusion-site polymerase chain reaction (AFS-PCR)

**DOI:** 10.1186/2162-3619-1-33

**Published:** 2012-11-09

**Authors:** Axel Weber, Sylvia Taube, Udo zur Stadt, Martin Horstmann, Knut Krohn, Jutta Bradtke, Andrea Teigler-Schlegel, Sabine Leiblein, Holger Christiansen

**Affiliations:** 1 Department of Pediatric Oncology, Hematology and Hemostaseology, Children’s Hospital, University of Leipzig, Leipzig, Germany; 2 Department of Human Genetics, University Hospital Giessen & Marburg, Giessen, Germany; 3 Center for Diagnostic, University Medical Center Hamburg Eppendorf, Hamburg, Germany; 4 Research Institute Children’s Cancer Center Hamburg and Clinic of Pediatric Hematology and Oncology, University Medical Center Hamburg, Hamburg, Germany; 5 IZKF Leipzig, University of Leipzig, Leipzig, Germany; 6 Oncogenetic Laboratory - Department of Pediatric Hematology/Oncology, Justus Liebig-University, Giessen, Germany; 7 Department of Hematology/Oncology, University of Leipzig, Leipzig, Germany

## Abstract

The amplification of putative oncogenes is a common finding within the genome of various cancer types. Identification and further targeting of specific junction sites within the sequence of genomic amplicons (amplicon fusion sites, AFS) by PCR (AFS-PCR) is suitable for quantification of minimal residual disease (MRD). This approach has recently been developed and described for *MYCN* amplified neuroblastomas. To compare AFS-PCR directly to routinely used MRD diagnostic strategies, we mapped the amplified genomic regions (ampGR) of an iAMP21-amplicon in high resolution of a patient with acute lymphoblastic leukemia (ALL). Successfully, we established AFS-PCR covering junction sites between ampGR within the iAMP21-amplicon. Quantification of MRD by AFS-PCR was directly comparable to IgH/TCR based real time quantitative PCR and fluorescence activated cell sorting (FACS) analysis in consecutive bone marrow (BM) specimens. Our data give an additional proof of concept of AFS-PCR for quantification of MRD. The method could be taken into account for ALL patients with genomic amplifications as alternative MRD diagnostic, if no or qualitatively poor Ig/TCR-PCRs are available.

## Background

Recently, we described a strategy for developing tumor cell specific PCRs for *MYCN* amplified neuroblastomas, using junction sites (amplicon fusion sites, AFS) of amplified genomic regions (ampGR) as template (AFS-PCR) [1]. All ampGR and thus, all AFS identified were absolute tumor cell specific and unique for each patient. AFS-PCR was highly sensitive and uncovered one tumor cell out of 10^6^ - 10^7^ control cells. We concluded this method is suitable for MRD quantification of *MYCN* amplified neuroblastoma. We furthermore hypothesized AFS-PCR might not only be limited to neuroblastoma, but transferable to other cancer types, provided that the individual tumor cells harbour ampGR.

The detection and quantification of minimal amounts of residual or recurrent leukemic blasts significantly improved therapy management for adult and childhood acute lymphoblastic leukemia (ALL) patients [2-4]. Routinely, rearrangements in immunoglobulin-chain (Ig) or T-cell receptor genes (TCR) serve as template for the design of tumor cell specific PCRs (Ig/TCR-PCR) [4-7]. Amplification of a part of the long arm of chromosome 21 including the *AML1/RUNX1* gene (iAMP21) occurs in 1-2% of ALL [8,9].

Bone marrow specimens from initial diagnosis and subsequent time points of an ALL patient with iAMP21 were used to directly compare AFS-PCR to routinely used MRD diagnostic strategies and to give additional proof of concept for AFS-PCR as a method for tumor cell detection, not only for neuroblastoma.

## Results

**Figure 1:**
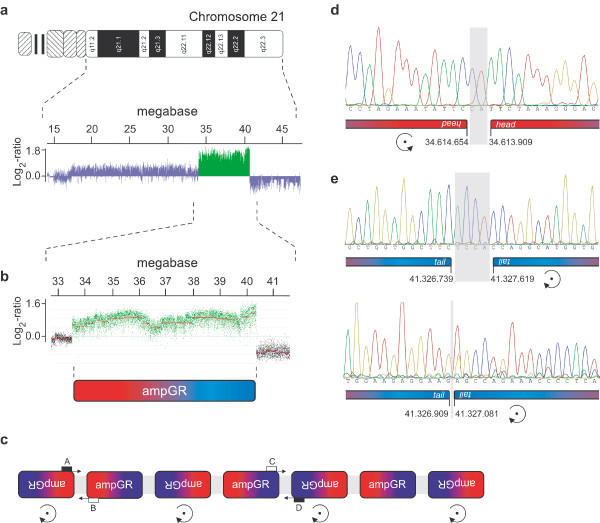
**Mapping of the ampGR on chromosome 21 and identification of the AFS sequences.** (**a**) Ideogram of chromosome 21 and corresponding data of the Affymetrix whole genome array. The amplified genomic region (ampGR) of chromosome 21 is highlighted in green. Data is presented as Log_2_-ratios of internal normalized signal intesities. (**b**) Data of the high resolution tiling array (NimbleGen). The relative copy number for each printed oligonucleotide are presented as fluorescent intensities of the Cy5 labelled test-DNA (cell lines) normalized to the Cy3 labelled reference-DNA (healthy human female) (Log_2_-ratio). The mean signal intensity value of the continuous genomic region was calculated by the SignalMap-Software and is indicated by the red line. The ampGR is highlighted in green. (**c**) Model of the architecture of the *AML1/RUNX1* amplicon. AmpGR are subsequently joined together in head-to-head and tail-to-tail orientation, resulting in tumor cell specific AFS. Primer for PCR are displayed as open or filled squares. (**d**) Chromatogram of the sequenced junction site of the head-to-head AFS-PCR-amplimer. (**e**) Chromatograms of the sequenced junction sites of the tail-to-tail AFS-PCR-amplimer.

Multiple copies of *AML1/RUNX1* had been identified by fluorescence-in-situ-hybridization (FISH) in a 9 year and 10 months old patient with precursor-B ALL. We confirmed *AML1/RUNX1* to be part of a large amplified genomic region of chromomosome 21 (iAMP21) and excluded coamplified regions or additional ampGR on other chromosomes by whole genome Array (Affymetrix Cytogenetics Whole-Genome *2.7M* Array) (Figure 1a). Besides iAMP21, several deletions were identified, with most relevant mapping to chromosomes 7p12.1-2 (including *IKZF1*), 11q23.3 (disrupting *MLL*), 12q13.12 and 12q24.11. (The deleted regions are quoted in detail in the Additional file 1: Table S1.) Exact mapping of the iAMP21 ampGR borders telomeric and centromeric to *AML1* was performed by high resolution Tiling-Array (HR-TA) of chromosome 21 (Human-CGH-385K-Chromosome-21 Tiling Array (Roche, NimbleGen)) (Figure 1b). Based on the array data the centromeric border of the iAMP21 ampGR was estimated between bases 34.613.680 and 34.615.721 (genomic annotation: GRCh37/hg19) and the telomeric border was estimated between bases 41.324.261 and 41.327.034. Overall, the ampGR covered 6.7Mb of genomic sequence. A list of amplified genes within the described region is given in Additional file 1: Table S1.

The first and the last 1000 bp of the amplified sequence of the iAMP21 were virtually fused to simulate junction sites between the subsequent copies of the ampGR. Thereby, we took all possible junction sites into account resulting from either head-to-tail or head-to-head and tail-to-tail orientation of the subsequent ampGR sequences. The junction site bridging AFS-PCRs were designed as previously described [1]. Interestingly the ampGR within the iAMP21-amplicon were found exclusively arranged head-to-head and tail-to-tail (Figure 1c), in contrast to the investigated *MYCN*-amplicons in primary neuroblastomas, in which all ampGR had been found in head-to-tail orientation [1]. Using different pairs of primer, two different tail-to-tail orientated AFS were identified. Although the ampGR were joined either in head-to-head or in tail-to-tail orientation, the junction sites were not found to be direct mirror planes at the single base-pair level. The subsequently joined ampGR showed different extents to their borders of about 745bp at the head-to-head junction and 880bp and 175bp, respectively at the tail-to-tail junctions. We found two additional bases (CA) inserted in the centromeric head-to-head AFS between the subsequent copies of the ampGR (Figure 1d), and four additional bases (TCCA) inserted in one of the two telomeric tail-to-tail AFS between the subsequent copies of the ampGR (Figure 1e). The second telomeric tail-to-tail AFS identified was a direct fusion of two subsequent ampGR without additional inserted nucleotides.

**Figure 2:**
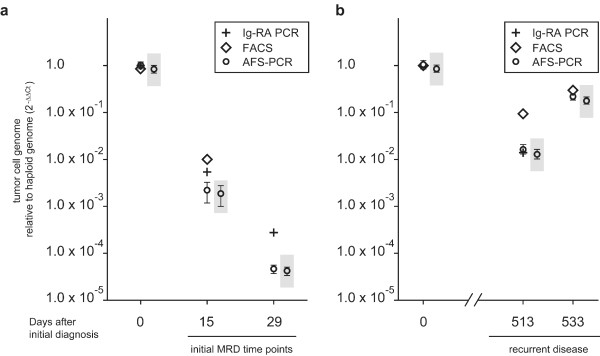
**Comparison of MRD values to different time points during therapy estimated by FACS-analysis, routine IgH/TCR-qPCR and AFS-PCR.** (**a**) Calculated tumor cell numbers within the bone marrow from the day of the initial diagnosis, day 15 and day-29 respectively. (**b**) Calculated tumor cell numbers within the bone marrow from the day of the initial diagnosis and the two bone marrow aspirates at day 513 and day-533 respectively. For AFS-PCR tumor cell contents of the different bone marrow aspirates are quantified according to the 2^-ΔΔCt^ calculation method. Each PCR was performed in triplicate. Mean values and standard deviations are indicated by dots and horizontal bars, respectively.

Because of the close proximity of the two different tail-to-tail AFS, it was not possible to design qPCR assays suitable for a valid quantification, as no suggested primer combinations resulted in a single specific amplimer. In contrast, the head-to-head-AFS served as a good template and AFS-qPCR was designed based on the exact AFS sequence. We quantified MRD in consecutive BM samples using the 2^-ΔΔCt^ Method as described in the material and methods section. Strikingly, we found the detected relative values of leukemic blasts by AFS-qPCR well comparable to the amounts of leukemic blasts detected by FACS or routine IgH/TCR-qPCR, respectively for most of the investigated time points (Figure 2**,** Additional file 2: Table S2**)**.

The blast count in the bone marrow at the initial day of diagnosis was estimated to be 85% by routine FACS analysis. Relative PCR quantification might be corrected to this value as described in the material and methods section**.** Corrected data are given in the gray-shaded boxes in Figure 2 and in separate columns within Additional file 2: Table S2.

## Discussion

Our data show that AFS-PCR is a suitable method for quantification of MRD of tumor cells with ampGR and that it is not limited to neuroblastoma with *MYCN*-amplification. Despite the almost equal quantification results of AFS-PCR compared to FACS and IgH/TCR-qPCR at days 513 and 533, respectively, the data at day 15 and 29 differ between 5 and 10 fold MRD count. These differences might be explainable at least in part by the different quantification procedures. As described, quantification of MRD by AFS-PCR was calculated in relation to a control gene (Inhibin-Beta-B; *INHBB*). This is in contrast to quantification of IgH/TCR-qPCR by comparing the PCR values of the subsequent bone marrow samples in relation to a serial dilution of DNA from leukemic blasts from the initial diagnosis into control DNA [6,7]. *INHBB* was chosen as control gene in this case of ALL because we have had the information about a numerically diploid karyotype and exclusion of *INHBB* beeing part of a small copy number variation (CNV) by whole-genome SNP Array in the leukemic blasts. However, although optimisation of qPCR, conditions for the relative quantification of the AFS-PCR against *INHBB* was performed accurately and although control *INHBB*-PCR and AFS-PCR both showed good efficiencies between 10^-1^ and 10^-5^ dilution steps (Additional file 3: Figure S1), it is not possible to exclude, that minor differences in PCR efficiencies could have influenced the quantification for day 15 and 29, respectively.

From the days 15 and 29 only a very small amount of dried bone marrow was obtainable for DNA isolation for AFS-PCR. Because of the low cell count to these time points, the whole specimens except a few bone marrow smears were used for routine diagnostics before. Indicating the low input DNA into AFS-qPCR, the Ct-values for the *INHBB* and the AFS-fragment, respectively, were significantly higher compared to those from the day of initial diagnosis or day 513 and day 533 (Additional file 4: Figure S2). The possibility to quantify MRD from such low amounts of input DNA indicates one great advantage for the relative quantification to a control gene, as with AFS-PCR, over the serial dilution method, which depends on an appointed amount of input DNA. However, with respect to differences in PCR efficiancies, the very low amount of input DNA in AFS-qPCR might also have a part in the lower MRD quantification values calculated for day 15 and day 29 as compared to IgH/TCR-qPCR.

Another advantage of AFS-PCR is the absolute specificity for the tumor cells in contrast to IgH/TCR-qPCR. In the latter, the tumor cell specificity varies, dependent on the degree of difference in the rearranged parts of the Ig/TCR genes of the leukemic blasts compared to untransformed B- and T-cells. It is known that the composition of the subpopulations of mononuclear white blood cells change during therapy, influencing the background signal and thereby the quantitative range of IgH/TCR-qPCR, most notably in samples with a low blast count [7]. In contrast, based on the specific sequences to both side of the AFS, it was possible to design AFS-PCR without a significant background for most of the neuroblastoma patients investigated [1] and the head-to-head junctions of the iAMP21 presented here (Additional file 4: Figure S2a).

Comparable to amplification of *MYCN* in childhood neuroblastomas, iAMP21 including *AML1/RUNX1* is discussed to be an initial event in the development of leukemic blasts comprising this genomic lesion, and no information exists that AFS change over time within these cells [8]. It is well known that in some cases, tumors or leukemias arise out of more than one tumor cell clone. However, in such cases not only AFS-PCR but all specific MRD methods are hampered to monitor the course of the disease exactly, focussing only on one tumor cell clone.

Transferring AFS-PCR to a patient with ALL, we can give additional proof of concept for the usefulness of targeting junction sites within genomic amplicons for MRD quantification. Furthermore, by direct comparison to standard IgH/TCR-qPCR and FACS analysis we can describe AFS-PCR as highly tumor cell-specific and sensitive for MRD quantification.

A major advantage of AFS-PCR over other methods for MRD quantification is the possibility of reliably detecting ampGR in primary tumor or bone marrow specimens, even with low tumor cell content. However, given by the dependency on AFS-sequences, the diagnostic algorithm is limited to patients with malignancies harbouring at least one detectable genomic amplification. Like for all PCR based methods, it might be challenging in some cases to design proper AFS-qPCR in time, to be of use in routine MRD diagnostics. Designing AFS-PCR, it is important to consider all possible orientations of the subsequent ampGR like head-to-tail, as reported for all investigated neuroblastomas or head-to-head and tail-to-tail as shown exemplarily with the ALL-patient described.

## Conclusion

Targeting AFS within iAMP21 in a patient with precursor-B-ALL we can show that quantification of MRD using AFS-PCR is comparable to other, routinely used techniques like FISH and Ig/TCR-PCR. The comparison of a new method with well-established techniques is an important keystone in a proof of concept and for MRD diagnostics almost exclusively possible with ALL. Although, AFS-PCR based on iAMP21 is potentially available for only 1-2% of patients with ALL, the method could be taken into account for these patients as alternative MRD diagnostic, if no or qualitatively poor Ig/TCR-PCRs are available.

It is without doubt, that, in future, next generation sequencing (NGS) of cancer genomes will play a major role in the diagnostic strategy for cancer patients, as it provides the possibility not only to identify genomic rearrangements on sequence level important for individual risk estimation or as possible targets for a personalized therapy, but also for use as MRD marker [10]. However, at present NGS of whole genomes is still expensive and time-consuming and thus, not suitable for a broad use in routine diagnostics. Enrichment strategies of prior defined target regions such as ampGR and analysis of multiple pooled specimens could be an alternative strategy to make MRD diagnostics available for a bigger number of patients with different types of cancer and to make it possible to estimate the relevance of MRD for a majority of malignancies.

## Material and methods

### Patient

We studied primary bone marrow specimens from a 9 year and 10 months old female patient with pre-B-ALL. At time of initial diagnosis the cytogenetic analysis revealed a diploid karyotype (46,XX). Absence of the FISH signal for the proximal part of one MLL gene suggested a deletion in this genomic region. Neither a typical translocation to *MLL* (*MLL/AF4, MLL/AF9, MLL/ENL*) nor *BCR/ABL* and *TEL/AML1* were found. Based upon cytogenetical findings and adequate response to the induction therapy (MRD values at day 15 below 10^-2^ and at day 29 below 10^-3^), the patient was stratified into the “standard-low-risk” treatment group and treated according to the german COALL-07-03 protocol with informed consent for therapy and study procedures [11]. (The study was reviewed by the Ethics committee of the physicians’ board (City of Hamburg) No. WF-50/08.) During maintenance therapy the patient’s condition worsened and rising MRD values were noticed (from day 513 after initial diagnosis). *AML1/RUNX1-*amplification was detected again in recurrent leukemic blasts. Therapy was intensified according to the german ALL-BFM-Rez protocol. In spite of consequently administered supportive medication, the patient suffered from a fulminant fungal infection and died from the disease 6 weeks later.

### Fluorescence in-situ hybridisation (FISH)

For FISH analysis specific probes were used for the *TEL/AML1* fusion gene (Vysis LSI ETV6(TEL)RUNX1(AML1) ES DUALColor Translocation Probe Set), the *MLL* gene (Vysis LSI MLL Dual Color, Break Apart Rearrangement Probe) and the *BCR/ABL* fusion gene (LSI BCR/ABL ES Translocation Probe Set) (all probes obtained from Abbott Laboratories, Abbott Park, IL). FISH was carried out according to the manufacturer’s instructions. Chromosomes were counterstained with 4,6-diamidino-2-phenylindole (DAPI). Digital imaging and documentation were performed employing a Zeiss Axio Imager.Z2 fluorescence microscope (ZEISS, Jena, Germany) equipped with an Isis image analysis system (Metasystems, Altlussheim, Germany). G-band-like images were generated by software-mediated conversion of DAPI staining into black and white. For each sample 100 nuclei were analysed.

### Fluorescence activated cell sorting (FACS)

Immunophenotyping of diagnostic samples was performed according to standardized protocols for monoclonal antibody staining. Fluorochrome conjugated antibodies were purchased from Becton Dickinson (BD, San José, CA, USA): CD45(2D1), CD14(MΦP9), CD10(W8E7), CD20(L27), CD34(8G12), CD13(L138), CD33(P67.6), CD22(S-HCL-1), kappa(TB28-2), lambda(1-155-2); Beckmann-Coulter: CD19(J4.119), CD24(ALB9), TdT(Pool); and Dako (Denmark): CD79a(HM57), Anti-IgM [F(ab)_2_](polyclonal rabbit). Flow cytometry analyses were performed within 24 h from collection using a FACS-Calibur (BD) and the data were processed using Cell Quest pro software. CD10 and CD19 costaining of leukemic blasts (CD10^bright^/CD19+) of the patient investigated in this study was used for MRD quantification. For MRD analysis at least 10.000 cells were acquired for each antibody combination.

### DNA isolation

Isolation of genomic DNA was performed from bone marrow specimens using the DNA-Blood and Tissue Kit (QIAGEN). DNA quality and concentration was measured spectrometrically (Nanodrop, Amersham). After the beginning of chemotherapy the cell count within the bone marrow samples decreased rapidly. For the days 15 and 29 all bone marrow specimens had been used for routine diagnostics (FACS analysis and IgH/TCR-qPCR) without any material left. The only source for DNA isolation for the two days, were cells scratched from dried bone marrow smears.

### Whole genome microarray analysis

100ng of genomic DNA isolated from bone marrow of the day of initial diagnosis was hybridized to a Cytogenetics Whole-Genome *2.7M* Array. The array was processed according to the instructions of the manufacturer using the Cytogenetics Reagent Kit (Affymetrix, Santa Clara, CA, USA). Chromosome-Analysis-Suite (Version: CytoB-N1.2.0.232 (r2480) / NetAffx Build 31 (hg19)) was used for analysis of the data.

### High resolution tiling array (HR-TA)

5μg of genomic DNA isolated from bone marrow of the day of initial diagnosis was hybridized to a Human-CGH-385K-Chromosome-21 Tiling Array (Roche, NimbleGen) according to the manufacturer’s conditions. The average resolution was one DNA-oligonucleotide every 70 bp. Hybridization of the Tiling Array was performed at ImaGenes (Details to the DNA quality criteria and the hybridization methods are available from ImaGenes, Berlin, Germany (http://www.imagenes-bio.com)). The DNA of the ALL specimen was Cy5-labeled whereas the control DNA was Cy3-labeled. SignalMap© Software (Ver. 1.9; NimbleGen) was used for the analysis of the HR-TA data. (Bp-coordinates cited in this study were taken from UCSC Genome Bioinformatics Database, Assembly February 2009 (NCBI37/hg19)).

### Primer design

All primers for standard PCR and RQ-PCR were designed using OLIGO Primer analysis software (Ver. 6.41; Molecular Biology Insights, Inc.). The sequences of primers used are:

#### AFS-fragments for Sanger-sequencing

1) head-to-head-AFS:

primer1: TGAACAGGCAACAGTCGTT, primer2: TCCCAGGTTCATGCCATTCTCCT

2) tail-to-tail-AFS-1:

primer1: GGTCGTTAAGCAGCCAATGA, primer2: TGTTGCCCAGACTGGAGT

3) tail-to-tail-AFS-2:

primer1: CAATGTTAGAGCCCAGTG, primer2: TGTCAGATTGGCCTCGTA

#### AFS-qPCR

1) *INHBB*:

primer1: AGTGTGTTTCCCCCATTGCCT, primer2: TCACACTGCACGTCTAGGTT

2) head-to-head-AFS:

primer1: AATGTCCTCAGAGGCAATTGTCCA, primer2:TGAACAGGCAACAGTCGTT

### AFS-PCR and sequencing

The PCR for validation of the virtual AFS was performed under standardized conditions. We used the QIAGEN HotStarTaq-Plus PCR Mastermix (12.5μl), 200ng DNA, 2 Primer at a final concentration of [1pMol] each, 1.5μl DMSO (SIGMA) and Aqua-dest. (Braun) ad 25μl. Cycler conditions were: 5 minutes initial denaturation at 95°C followed by 38 cycles with 20 seconds at 95°C, 20 seconds at 57°C and 40 seconds at 72°C. DNA from human placenta tissue served as negative control (cntr.). Electrophoresis was performed in 1%-3% agarose gels dependent on the PCR fragment length. Gels were stained with ethidium bromide and bands were visualized under UV light (Image Master VDS (Pharmacia)). Bands of estimated length were excised from the gel and PCR fragments were isolated using the QIAGEN gel extracting kit following the manufacturer’s instructions. Each AFS-fragment was sequenced from both sides using a BigDye Terminator 3.1 Ready Reaction Cycle Seq. Kit (Applied Biosystems) following the manufacturer’s instructions. We used a 16 Capillary Sequencer Genetic Analyzer 3100 from Applied Biosystems. 250ng of DNA and 10pmol of one primer were put in one sequencing reaction.

### Real-time AFS-PCR

Realtime-PCR (RQ-PCR) conditions were: SIGMA SYBR-GreenJumpStart-Taq Ready Mix (12.5μl), 200ng DNA, 2 Primer at a final concentration of [1pMol] each, 1.5μl DMSO, 2.5μl and Aqua-dest. ad 25μl. Cycler conditions were: 5 minutes initial denaturation at 95°C followed by 44 cycles with 20 seconds at 95°C, 20 seconds at 54-58°C and 40 seconds at 70-72°C (depending on individual primer binding conditions). All real-time-PCR were performed on a BIORAD iQ5-Cycler. Each real-time PCR, including the internal control (Inhibin-beta-b (*INHBB*)) was performed in triplicate. Ct-Values, melt curves and PCR efficiencies are displayed in Additional file 4: Figure S2 and Additional file 3: Figure S1 respectively.

### Ig/TCR based detection of MRD

PCR studies for MRD analysis were performed with IgH and TCR gene rearrangements as targets. Junctional regions of clonal products were sequenced directly and patient specific junctional regions were identified for generation of allele specific PCR primers. Biclonal or biallelic products were cloned using the TOPO-TA cloning kit (Invitrogen) and then processed adequately for generation of suitable patient specific primers. Subsequently PCR-MRD targets were tested for specificity and sensitivity to reach a sensitivity and a quantifiable range of 1 × 10^-4^ for at least two targets. Realtime quantitative PCR (RQ-PCR) was performed and interpreted according to the guidelines developed within the European Study Group for MRD detection in ALL (ESG-MRD ALL) [7]. In detail, sequence specific TaqMan probes were used on a LC480 machine (Roche). Tenfold serial dilutions of diagnostic DNA were prepared in pooled peripheral blood DNA extracted from at least five healthy donors. Quantification was performed using this standard curve and triplicates of follow-up samples including 500 ng DNA in each reaction. In the initial diagnostic material the following leukemia specific targets were detected: the following leukemia specific targets were detected: **Dd2-DD3** (QR 1 × 10^-4^; QS 5 × 10^-4^); **Vk1-Kde** (QR 5 × 10^-4^; QS 5 × 10^-4^); VγI-Jg1.1; VγI-Jg1.3; Vβ2-Jβ2.3. Targets in bold letters with a quantifyable range of at least 5 × 10^-4^ were chosen for quantification of the follow up samples (QR means the quantifyable range and QS correlates with the sensitivity of each target, both evaluated according the guidelines of the ESG-MRD-ALL group, as described previously in more detail [7].

### Statistics

The relative amounts of *AML1/RUNX1* amplified leukemia cells within the different bone marrow samples, investigated, were calculated using the 2^-ΔΔCt^ method [12]. Therefore, the Ct values of the specific AFS fragments were normalized to the corresponding Ct values of the *INHBB* control PCR fragments. Each resulting ΔCt value was further normalized to the median ΔCt of the *AML1/RUNX1* amplified leukemia DNA from the initial day of diagnosis. This calculation resulted in a triplicate of 2^-ΔΔCt^ values for each specimen, investigated. RQ-PCR data in Figure 2 present the mean and standard deviation of these 2^-ΔΔCt^ triplicates.

To correct the Ct-value of the specific AFS-PCR fragment of the primary tumor DNA to the tumor cell content assessed by FACS analysis we used the following calculation:

(1) $$\text{AFS}-{\text{Ct}}_{\left(\text{corrected}\right)}=\text{AFS}-{\text{Ct}}_{\left(\text{native}\right)}+{\text{Log}}_{2}X$$

(*X* being the relative tumor cell content of the primary tumor specimen, e.g. 0.8 for 80%). All other Ct values stayed unchanged. ΔCt values were than calculated in relation to the corrected ΔCt value of the primary tumor specimen according to the 2^-ΔΔCt^ method (Data points in grey shaded boxes in Figure 2 and separate data in Additional file 2: Table S2).

## Abbreviations

AFS: (Amplicon fusion site); ampGR: (Amplified genomic region); BM: (Bone marrow); CGH: (Comperative genomic hybridisation); FACS: (Fluorescence activated cell sorting); FISH: (Fluorescence *in-situ* hybridisation); HR-TA: (High resolution tiling array); Ig: (Immunoglobulin chain gene); LN: (Lymph node); MRD: (Minimal residual disease); qPCR: (Quantitative real-time polymerase chain reaction); TCR: (T-cell receptor).

## Competing interests

The authors declare that they have no competing interests.

## Authors’ contributions

AW, idea, experimental design and bioinformatical work; AW and ST performed the AFS-PCR experiments; UzS and MH performed the Ig-RA PCR experiments; KK performed the whole genome microarray analysis; JB and AT-S performed the FISH analyses; SL performed FACS analyses; HC coordinated and supervised the project; AW wrote the manuscript and prepared the figures. All authors read and approved the manuscript.

## Supplementary Material

Additional file 1 **Table S1.** Amplified and deleted chromosomal regions and the genes covered by these aberrations in the investigated case of ALL identified by whole genome array (Affymetrix Cytogenetics Whole-Genome *2.7M* Array). Click here for file

Additional file 2 **Table S2.** Comparison of the calculated MRD values estimated by FACS analysis, IgH/TCR-qPCR and AFS-PCR. **(a)** Blast count in bone marrow samples from initial diagnosis, day 15 (d15) and day 29 (d29) after the beginning of therapy. **(b)** Blast count in bone marrow samples from initial diagnosis and recurrent disease at days 513 (d513) and 533 (d533). Click here for file

Additional file 3 **Figure S1.** Ct-Values (left column) and melting curves (right column) of the *INHBB* and the AFS-PCR fragments. **(A)** First row: *INHBB*-PCR and second row: AFS-PCR for detection of MRD values on day 513 (blue) and day 533 (red) including a AFS-negative control DNA (human placenta) (purple). **(B)** First row: *INHBB*-PCR and second row: AFS-PCR for detection of MRD values on day 15 (orange) and day 29 (light blue). Click here for file

Additional file 4 **Figure S2.** PCR efficiencies tested by a 1:10 dilution series of DNA isolated from bone marrow at time of initial diagnosis. **(A)** Ct-values, melting curve and calculated efficiency of the *INHBB*-PCR. **(B)** Ct-values, melting curve and calculated efficiency of the AFS-PCR. PCR efficiencies were calculated by the BioRad IQ5-Software (Version 2.1.97.1001). Click here for file
