# Supplementary material for: Quantification of minimal residual disease (MRD) in acute lymphoblastic leukemia (ALL) using amplicon-fusion-site polymerase chain reaction (AFS-PCR)
Source: Exp Hematol Oncol. 2012 Nov 9;1:33. doi: 10.1186/2162-3619-1-33 (PMC3518178; doi:10.1186/2162-3619-1-33)
Supplement: Additional file 1 — Table S1. Amplified and deleted chromosomal regions and the genes covered by these aberrations in the investigated case of ALL identified by whole genome array (Affymetrix Cytogenetics Whole-Genome 2.7M Array). [file 2162-3619-1-33-S1.pdf]

Additional Table 1

Human Genome Assembly GRCh37 hg19

	begin	end	genes
amplified genomic region (ampGR) **Chr21q**	34,537,706	41,326,577	*IFNAR2, IL1ORB, IFNAR1, IFNGR2, THEM5OB, DNAJC28, GART, SON, DONSON, CRYZL1, ITSN1, ATP50, MRPS6, SLC5A3, KCNE2, FAM165B, KCNE1, RCAN1, CLIC6,*<br><br>***AML1/RUNX1****,*<br><br>*SETD4, CBR1, CBR3, DOPEY2, MORC3, CHAF1B, CLDN14, SIM2, HLCS, DSR6, PIGP, TTC3, DSCR3, DYRK1A, KCNJ6, DSCR4, KCNJ15, ERG, ETS2, PSMG1, BRWD1, HMGN1, WRB, LCA5L, SH3BGR, B3GALT5, IGSF5, PCP4*
Deletion **Chr21q**	41,376,587	47,917,559	http://genome.ucsc.edu/cgi-bin/hgTracks?db=hg19&position=chr21:41376587-47917559
Deletion **Chr7p**	50,043,337	51,004,732	*ZPBP, C7orf72,* ***IKZF1/Ikaros****, FIGNL1, DDC, GRB10*
Deletion **Chr7q**	126,030,725	159,118,443	http://genome.ucsc.edu/cgi-bin/hgTracks?db=hg19&position=chr7:126030725-159118443
Deletion **Chr11q**	117,719,425	118,324,674	*FXYD6, FXYD2, TMPRSS13, IL10RA, TMPRSS4, SCN4B, AMICA1, MPZL3, CD3E, CD3D, CD3G, UBE4A, ATP5L,* ***MLL***
Deletion **Chr12q**	49,556,573	50,741,639	*TUBA1A, TUBA1C, PRPH, TROAP, C1QL4, DNAJC22, SPATS2, KCNH3, MCRS1, FAM186B, PRPF40B, FMNL3, TMBIM6, NCKA5L, BCIN3D, FAIM2, AQP2, AQP5, AQP6, RACGAP1, ACCN2, SMARCD1, GPD1, CERS5, LIMA1, FAM186A*
Deletion **Chr12q**	111,068,175	111,859,722	*TCTN1, HVCN1, PPP1CC, CCDC63, MYL2, CUX2, FAM109A, SH2B3*
