# Supplementary material for: Quantification of minimal residual disease (MRD) in acute lymphoblastic leukemia (ALL) using amplicon-fusion-site polymerase chain reaction (AFS-PCR)
Source: Exp Hematol Oncol. 2012 Nov 9;1:33. doi: 10.1186/2162-3619-1-33 (PMC3518178; doi:10.1186/2162-3619-1-33)
Supplement: Additional file 2 — Table S2. Comparison of the calculated MRD values estimated by FACS analysis, IgH/TCR-qPCR and AFS-PCR. (a) Blast count in bone marrow samples from initial diagnosis, day 15 (d15) and day 29 (d29) after the beginning of therapy. (b) Blast count in bone marrow samples from initial diagnosis and recurrent disease at days 513 (d513) and 533 (d533). [file 2162-3619-1-33-S2.pdf]

additional Table 2 (d*ata according to Figure 2*)

a

					AFS-PCR data not corrected		AFS-PCR data corrected	
Days after initial diagnosis	FACS	FACS (relative value)	Ig-RA-PCR	Ig-RA-PCR (mean value)	$2^{-\Delta\Delta Ct}$ (AFS/INHBB)	mean value to initial 100% leukemic cells	$2^{-\Delta\Delta Ct}$ (AFS/INHBB)	mean value to initial 85% leukemic cells
Initial	85%	0,850000			0,878633	1,026357	0,746838	0,872404
					1,369200		1,163820	
					0,831238		0,706552	
d15	1%	0,010000	0,006074	0,005429	0,001579	0,002219	0,001342	0,001886
			0,004868		0,000823		0,000700	
			0,005346		0,004255		0,003617	
d29			0,000336	0,000278	0,000050	0,000047	0,000043	0,000040
			0,000269		0,000061		0,000052	
			0,000229		0,000029		0,000024	

b

					AFS-PCR data not corrected		AFS-PCR data corrected	
	FACS	FACS (relative value)	Ig-RA-PCR	Ig-RA-PCR (mean value)	$2^{-\Delta\Delta Ct}$ (AFS/INHBB)	mean value to initial 100% leukemic cells	$2^{-\Delta\Delta Ct}$ (AFS/INHBB)	mean value to initial 85% leukemic cells
initial	85%	0,850000			1,064370	1,001080	0,904715	0,850918
					0,986233		0,838298	
					0,952638		0,809742	
d513	10%	0,100000	0,020000	0,020000	0,010525	0,016341	0,008946	0,013890
					0,022097		0,018783	
					0,016402		0,013942	
d533	30%	0,300000			0,173139	0,219962	0,147168	0,186968
					0,233258		0,198270	
					0,253490		0,215466	
