# Supplementary figures and images for: Quantification of minimal residual disease (MRD) in acute lymphoblastic leukemia (ALL) using amplicon-fusion-site polymerase chain reaction (AFS-PCR)
Source: Exp Hematol Oncol. 2012 Nov 9;1:33. doi: 10.1186/2162-3619-1-33 (PMC3518178; doi:10.1186/2162-3619-1-33)

additional Figure 1

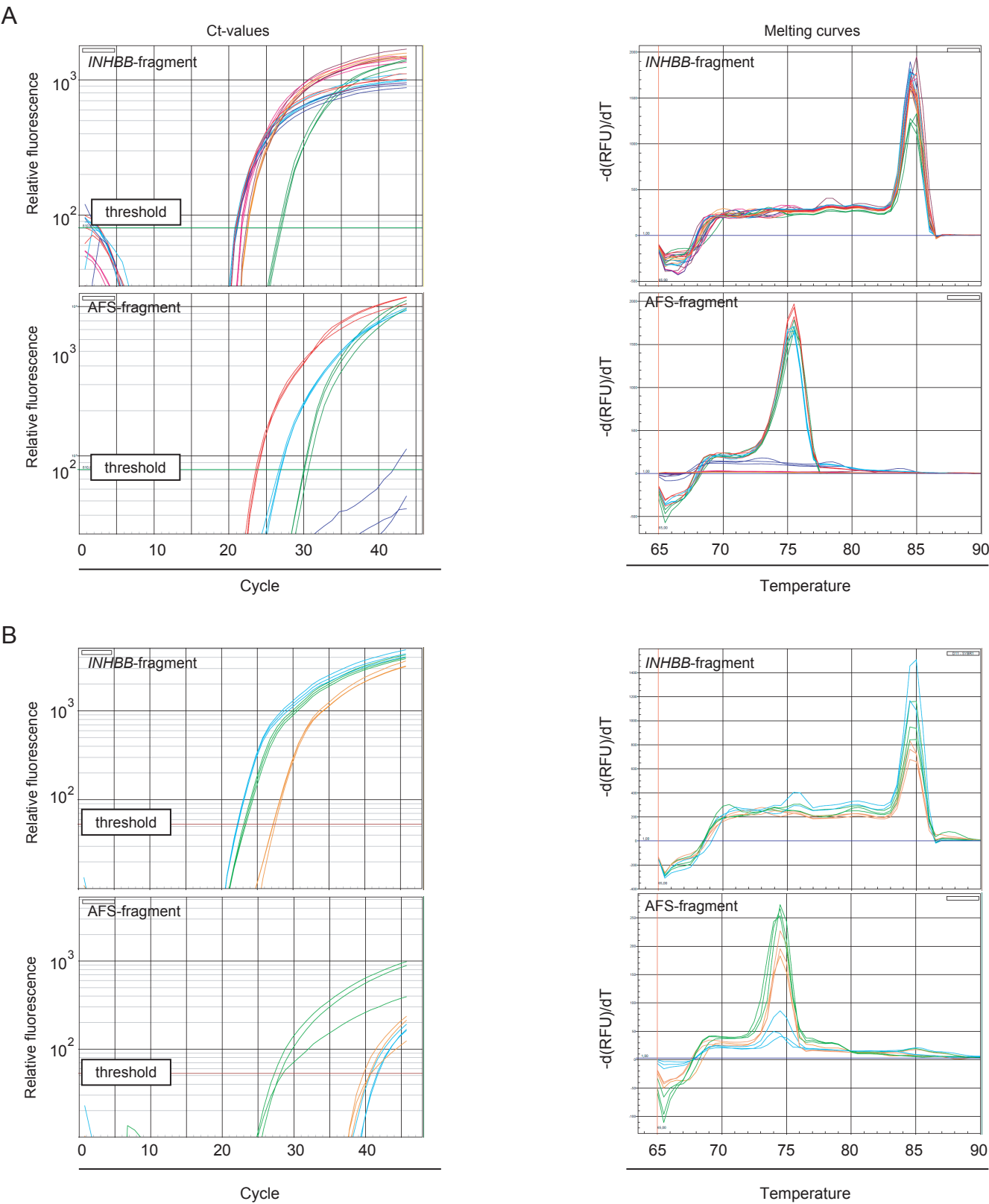

Supplement: Additional file 3 — Figure S1. Ct-Values (left column) and melting curves (right column) of the INHBB and the AFS-PCR fragments. (A) First row: INHBB-PCR and second row: AFS-PCR for detection of MRD values on day 513 (blue) and day 533 (red) including a AFS-negative control DNA (human placenta) (purple). (B) First row: INHBB-PCR and second row: AFS-PCR for detection of MRD values on day 15 (orange) and day 29 (light blue). [file 2162-3619-1-33-S3.pdf]

additional Figure 2

A

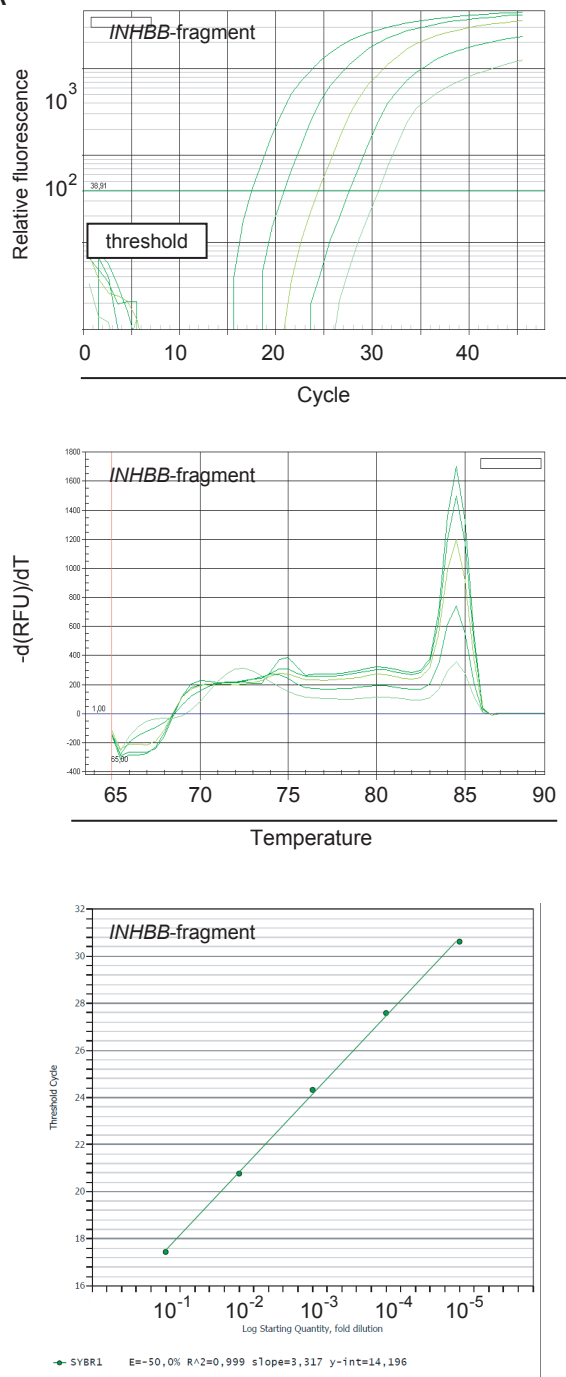

B

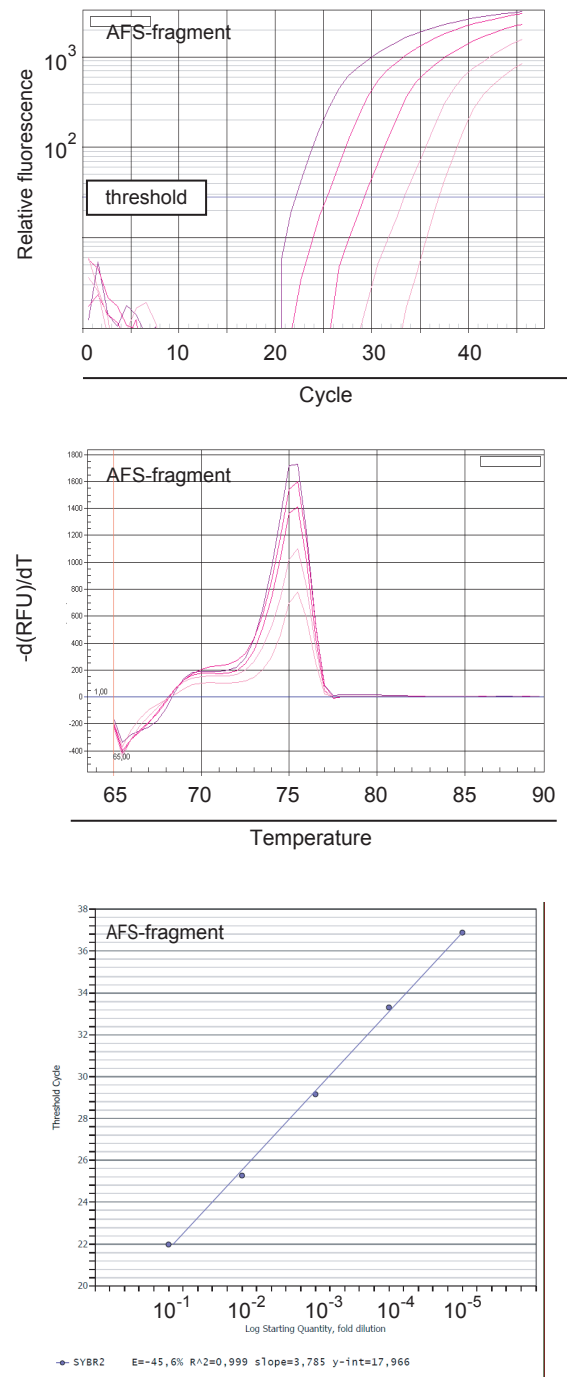

Supplement: Additional file 4 — Figure S2. PCR efficiencies tested by a 1:10 dilution series of DNA isolated from bone marrow at time of initial diagnosis. (A) Ct-values, melting curve and calculated efficiency of the INHBB-PCR. (B) Ct-values, melting curve and calculated efficiency of the AFS-PCR. PCR efficiencies were calculated by the BioRad IQ5-Software (Version 2.1.97.1001). [file 2162-3619-1-33-S4.pdf]
